# Peptide Receptor Radionuclide Therapy of Differentiated Thyroid Cancer: Efficacy and Toxicity

**DOI:** 10.1007/s00005-014-0318-6

**Published:** 2014-11-18

**Authors:** Rafał Czepczyński, Magdalena Matysiak-Grześ, Maria Gryczyńska, Maciej Bączyk, Anna Wyszomirska, Marek Stajgis, Marek Ruchała

**Affiliations:** 1 Department of Endocrinology, Poznan University of Medical Sciences, Przybyszewskiego 49, 60-355 Poznań, Poland; 2 Department of Radiology, Poznan University of Medical Sciences, Poznań, Poland

**Keywords:** Thyroid cancer, Peptide receptor radionuclide therapy, Efficacy, Toxicity

## Abstract

In rare cases of differentiated thyroid carcinoma (DTC), radioiodine treatment is no longer effective due to cell dedifferentiation. Targeting somatostatin receptors in DTC cells by radiolabelled somatostatin analogues could provide an alternative therapy option. The aim of this study was to evaluate safety and efficacy of peptide receptor radionuclide therapy (PRRT) in patients with advanced, non-iodine avid DTC. Eleven patients aged 47–81 years (median: 65 years) with a history of several courses of radioiodine therapy, increasing thyroglobulin (Tg) and negative whole body scan, were qualified to the study. After confirming receptor expression by somatostatin receptor scintigraphy, PRRT with yttrium-90 labelled analogue was initiated. Fractionated treatment protocol was used with four doses of ^90^Y-DOTA-TOC in 12-week intervals. Activity of each dose was 3.7 GBq (100 mCi). Of 11 patients, 5 died before receiving the fourth course of PRRT. In the remaining six patients, morphological response, evaluated 3 months after the last course using RECIST criteria showed partial remission (PR) in one patient, stable disease (SD) in two patients and progressive disease (PD) in three patients. Biochemical response based on Tg measurements before and after PRRT showed PR in one patient, SD in four patients and PD in one patient. Median survival was 21 months from the first course of PRRT. Only minor and transient hematological toxicity was observed in some patients. We conclude that PRRT is generally well-tolerated and may be a valuable option for some patients with radioiodine-refractory DTC.

## Introduction

Differentiated thyroid carcinoma (DTC) is the most common endocrine malignancy (Schlumberger and Pacini 2003). The standard treatment of DTC involves complete thyroidectomy followed by radioiodine treatment (RIT). Relatively high efficacy of this treatment regimen is expressed by high percentage of patients who achieved remission; 10-year survival rate of 80–99 % is reported by different centres (Leboulleux et al. 2005; Tsang et al. 1998). However, as in all malignant diseases, cases of DTC progression are not uncommon. Most frequently, regional lymph node metastases or local recurrence in the thyroid bed may occur. Distant metastases, usually in lungs and bones, are less common. Presence of distant metastases highly worsens patient’s prognosis. Ten-year survival rate decreases to ca. 40 % in patients with metastases, despite the use of still partially effective RIT courses (Schlumberger and Pacini 2003). Disease progression is often combined with the dedifferentiation of DTC cells; due to the mutation of the natrium–iodide symporter gene, the cancer cells lose their ability to accumulate iodine (Lazar et al. 1999). As the result, the whole body scans show no iodine accumulation in the known metastatic foci despite elevated concentrations of DTC marker, thyroglobulin (Tg). This clinical situation was recently named TENIS syndrome, i.e. thyroglobulin elevation, negative iodine scan (Silberstein 2011). The loss of iodine-avidity is associated with a more aggressive disease pattern (Sherman 2003). It is estimated that the problem of dedifferentiation affects approximately one-third of patients with disseminated DTC (Ma et al. 2005). Occurence of dedifferentiation is a critical point since it virtually excludes the only efficient treatment option—RIT. Other conventional treatment methods, i.e. surgery and external beam radiation therapy are not useful in case of widespread metastatic disease. On the other hand, chemotherapy that is usually applied in metastatic forms of other malignancies (as breast or ovarian cancer) was reported ineffective in DTC (Cooper et al. 2009; Santini et al. 2002).

In the 1990s, somatostatin receptors (SSTR) were identified in the cells of majority of endocrine malignancies (Lamberts et al. 1991; Reubi et al. 1987). Based on these findings, radiolabelled somatostatin analogues—ligands to the appropriate subtypes of SSTR were developed. Analogues labelled with indium-111 and technetium-99m are used for scintigraphic visualization of the disease foci and analogues labelled with gallium-68 are used for positron emission tomography (Gabriel et al. 2005; Krenning et al. 1993; Pettinato et al. 2008).

In addition to the wide range of diagnostic applications, radiolabelled somatostatin analogues have been also used for treatment purpose in the form of peptide receptor radionuclide therapy (PRRT). The analogues labelled with beta-emitters (yttrium-90 or lutetium-177) were found to provide encouraging results in patients with disseminated forms of neuroendocrine tumours (NET) (Bodei et al. 2004; Kunikowska et al. 2011). Data regarding the treatment possibilities of other endocrine malignancies, including DTC, are limited. There are, however, convincing data documenting utility of somatostatin analogues in the imaging of DTC (Gabriel et al. 2004; Stokkel et al. 2003). In face of lacking treatment possibilities for the patients with non-iodine avid DTC, PRRT could be an attractive option. It is expected that PRRT would deliver beta-emitting agent to the tumour cells using completely different uptake mechanism than in RIT. There is little data on the efficacy of PRRT in DTC. Therefore, this retrospective analysis has been performed with the aim of evaluation of feasibility, safety and efficacy of PRRT in patients with DTC.

## Materials and Methods

Presented study is a retrospective analysis of PRRT that was experimentally applied in patients with disseminated DTC in whom further courses of RIT were contraindicated due to deficient uptake of radioiodine.

Patients with DTC with non-iodine avid distant metastases were included in the study group. Following inclusion criteria were used to select the study cohort:

1. total thyroidectomy and at least three RIT courses;
2. elevated and increasing Tg both on l-thyroxin therapy and on thyroid-stimulating hormone stimulation within 3 months before study entry;
3. negative whole body scan after recent RIT;
4. imaging evidence of metastatic lesions with use of ultrasonography of the neck (US) and computed tomography of the chest and neck (CT);
5. no indications to surgery of metastatic foci of DTC;
6. positive SSTR scintigraphy performed in the last 2 months;
7. laboratory criteria: haemoglobin >10 g/dL, leucocytes >3.0 × 10^9^/L, platelets >100 × 10^9^/L, creatinine <1.2 mg/dL, glomerular filtration rate >30 mL/min;
8. no clinical signs of comorbidities increasing the risk of PRRT;
9. life expectancy of at least 6 months;
10. written informed consent to PRRT and to the study.

Protocol of this retrospective study was approved by the bioethical committee of our institution.

### Somatostatin Receptor Scintigraphy

**Fig. 1:**
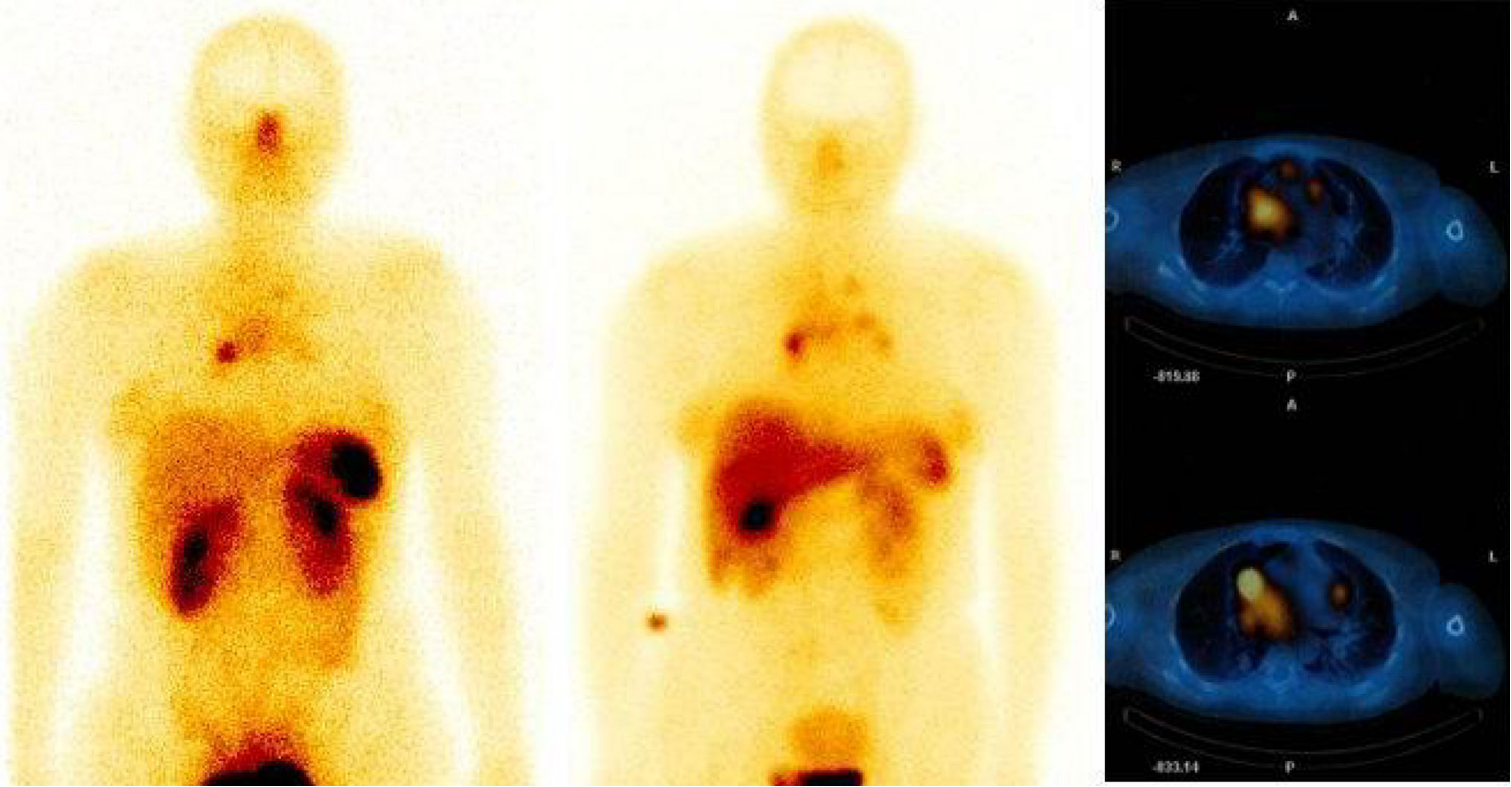
Peptide receptor scintigraphy using ^99m^Tc-HYNIC-TOC in a patient with mediastinal lymph node metastases of differentiated thyroid carcinoma (planar and SPECT/CT images)

Prior to PRRT, somatostatin receptor scintigraphy (SRS) was used to verify patient’s indications to the therapy. SRS was performed using technetium-labelled somatostatin analogue ^99m^Tc-EDDA/HYNIC-TOC (Tektrotyd manufactured by Polatom, Poland). Administered activity: 740 MBq. The images were obtained twice—3 and 24 h post injection using one of the dual-head gamma cameras: Varicam (Elscint, Israel) or Infinia Hawkeye-4 (GE Medical Systems, USA) equipped with low-energy all-purpose collimators. The early acquisition included also SPECT or SPECT/CT images of the neck and chest regions (Fig. 1). Images of SRS were evaluated by two experienced observers. SRS was regarded as positive if it showed foci of increased tracer accumulation corresponding with lesions detected with other imaging modalities (US, CT). In this sense, positive SRS was regarded as in vivo confirmation test for SSTR expression in the lesions.

### PRRT Protocols

A fractionated treatment protocol was used with four doses of ^90^Y-DOTA-TOC in 12-week intervals. Activity of each dose was 3.7 GBq (100 mCi). ^90^Y-DOTA-TOC was manufactured by Polatom (Poland). The preparation of ^90^Y-DOTA-TOC was administered intravenously over 20 min. To minimize radiation exposure of the kidneys, an intravenous amino acid solution, Nephrotect (Fresenius Kabi, Germany) was infused intravenously with the flow rate of 150 mL/h. The infusion was initiated 2 h before ^90^Y-DOTA-TOC infusion and continued over 6 h after ^90^Y-DOTA-TOC administration (Bodei et al. 2013). Altogether, the administered volume of amino acid solution was 1,000–1,400 mL.

Before each course of PRRT, as well as 3 months after the last course of PRRT, physical examination of the patient was performed, and following laboratory measurements were obtained: blood counts, creatinine, urea, transaminases, electrolytes, Tg and anti-thyroglobulin antibodies (aTg). These measurements (except Tg and aTg) were repeated 24 h and 3 weeks after ^90^Y-DOTA-TOC administration for the evaluation of toxicity. Final evaluation of laboratory data was performed 1 year after the last PRRT.

In addition, 3 months and 1 year after the last PRRT following imaging procedures were repeated: SRS, US of the neck and CT of the chest and neck. Imaging parameters were the same as at the initial evaluation.

### Evaluation of Response and Toxicity

Tumour response to PRRT was evaluated by the comparison of the lesion diameters between the initial and final US and CT. The measurements and interpretation of response was interpreted according to the Response Evaluation Criteria in Solid Tumours (RECIST) (Eisenhauer et al. 2009). According to these criteria, stable disease (SD) was defined as ≤30 % reduction or increase in the sum of the longest diameter of target lesion, progressive disease (PD) as ≥20 % increase in this diameter or if new lesions were noted. Patients were considered in partial response (PR) if at least 30 % reduction in the sum of the longest diameters of target lesions was observed.

Biochemical response was based on the comparison of Tg concentrations performed prior to PRRT and after the last course of the treatment. Using these biochemical criteria PR was defined as the reduction of Tg by at least 25 % and PD was noted if the increase of Tg by at least 25 % was measured. SD was defined as the relative change of Tg levels not exceeding 25 %. All presented Tg values were measured during suppressive l-thyroxine medication (de Keizer et al. 2003).

In case of remission (complete remission or PR), obtained results were confirmed using repeated US, CT and Tg measurements 4 weeks after the final evaluation (Eisenhauer et al. 2009).

Toxicity of PRRT was evaluated using Common Terminology Criteria of Adverse Events (CTCAE) version 4.0 (CTCAE 2010).

### Statistical Analysis

Continuous variables are expressed by their median and range. Probability of overall survival after first PRRT was estimated from Kaplan–Meier life tables. Value of *p* < 0.05 was considered to be statistically significant. Data analysis was performed using STATISTICA data analysis software system, version 10. www.statsoft.com.

## Results

Out of 16 patients in whom PRRT had been considered, 5 patients were disqualified: 3—due to low uptake of the tracer in the DTC foci at SRS and 2 other patients—due to laboratory contraindications (anaemia, granulocytopenia).

Eleven patients aged 47–81 years (median: 65 years) were qualified. The group consisted of nine female and two male patients. Five patients were diagnosed of follicular thyroid carcinoma (FTC), three patients had papillary thyroid carcinoma (PTC), and in the remaining three patients oxyphilic carcinoma (Hurthle cell thyroid carcinoma, HCTC) was diagnosed.

All the patients were previously treated with thyroidectomy and RIT (3–8 courses, median cumulative activity 3.1 GBq, 840 mCi). Due to the recurrence in the cervical region, six patients were treated surgically by the means of rethyroidectomy or cervical lymphadenectomy. In addition, one patient received external beam radiation therapy. The patients were referred to PRRT due to lung metastases (nine patients), bone metastases (three patients), local or lymph node recurrence (four patients). Before first PRRT, median Tg concentration was 242 ng/mL (range 32–524 ng/mL). Clinical data of the qualified patients are presented in Table 1.

**Table 1:** Summary of clinical data of the studied patients

No.	Sex	Age	Diagnosis	Stage	Previous therapy	Time since diagnosis (months)	Cumulative activity of radioiodine (GBq)	Tg (ng/mL) at baseline	Location of the disease foci
1	F	59	FTC	IVb	TT, RT, RIT	132	23.3	62	Lungs
2	F	60	HCTC	II	TT, RIT	133	26.6	295	Lungs
3	F	47	FTC	I	TT, L, RIT	66	53.3	52	Local recurrence, lungs
4	M	59	HCTC	IVc	TT, RIT	67	40.3	299	Lungs
5	F	76	HCTC	II	TT, RIT	106	27.7	185	Local recurrence, lymph nodes
6	F	70	FTC	IVc	TT, RT, XBR, RIT	87	25.5	524	Lungs, bones
7^a^	M	69	PTC	IVa	TT, L, RIT	61	21.0	309	Lymph nodes
8^a^	F	81	PTC	II	TT, RIT	73	35.5	186	Lungs
9^a^	F	77	FTC	IVa	TT, RT, RIT	109	22.2	456	Lungs, bones
10^a^	F	59	PTC	IVa	TT, L, RIT	159	30.0	32	Lungs, bones
11^a^	F	59	FTC	IVb	TT, RIT	96	27.7	500	Local recurrence, lungs

*F* female, *M* male, *FTC* follicular thyroid cancer, *PTC* papillary thyroid cancer, *HCTC* Hurthle cell thyroid carcinoma, *TT* total thyroidectomy, *RT* rethyroidectomy, *XBR* external beam radiation therapy, *L* lymphadenectomy, *RIT* radioiodine therapy

^a^Patients who died before completing PRRT

### Treatment Response

Five patients deceased before achieving the final course: two patients after first course, one patient after second course and two patients after third course of PRRT. The cause of death was directly related to the disease progression, except for the patient no. 7 who died due to pulmonary embolism. Subsequently, the total PRRT activity accounting for 14.8 GBq (400 mCi) in four courses was administered only to six patients. Thus, only this subgroup was eligible for the final response evaluation (patients no. 1–6).

Morphological and biochemical response to treatment in individual patients are presented in Table 2. Morphological response evaluated 3 months after the last treatment using RECIST criteria showed PR in one patient, SD in two patients and PD in three patients. Biochemical response based on Tg measurements showed PR in one patient (the same who had morphological PR), SD in four patients and PD in one patient. Median Tg (in patients no. 1–6 only) increased from 147 ng/mL at baseline to 173 ng/mL after completion of PRRT.

**Table 2:** Morphological and biochemical response

No.	Cumulative activity 90Y-DOTA-TATE(GBq)	Follow-up time since the first course of PRRT (months)	Tg (ng/mL) at baseline	Therapy response 3 months post therapy			Therapy response 1 year post therapy		
				Morphological	biochemical	Tg (ng/mL)	Morphological	Biochemical	Tg (ng/mL)
1	14.8	68	62	SD	SD	68	PD	PD	87
2	14.8	59	295	PR	PR	173	SD	PR	132
3	14.8	65	52	PD	PD	74	PD	PD	116
4	14.8	63^a^	299	PD	SD	313	PD	PD	>500
5	14.8	59	185	SD	SD	209	PD	PD	403
6	14.8	14	524	PD	SD	488	–	–	–
7	3.7	2^a^	309	–	–	–	–	–	–
8	11.1	11^a^	186	–	–	–	–	–	–
9	7.4	14^a^	456	–	–	–	–	–	–
10	11.1	9^a^	32	–	–	–	–	–	–
11	3.7	3^a^	500	–	–	–	–	–	–

*SD* stable disease, *PR* partial response, *PD* progressive disease

^a^Follow-up ended with patient’s death

The surviving subset of patients was followed up after completion of PRRT for 59–68 months from the first course of the therapy. Of these six patients, one died at 63 months post therapy initiation. The remaining five patients were reevaluated 1 year after completion of PRRT. In comparison to the evaluation at 3 months, four patients presented with PD, both in morphological and biochemical criteria. One patient showed signs of morphological SD and biochemical PR. It was the same patient (no. 2) who had responded with PR already at the early evaluation 3 months post therapy. The median overall survival was 21 months from the first course of PRRT.

To verify the impact of the disease stage on the treatment efficacy, the whole study group (11 patients) was divided into two subgroups according to the Tg concentration prior to the first PRRT: group 1 with Tg up to 150 ng/mL (*n* = 5) and group 2 with Tg above 150 ng/mL (*n* = 6).

**Fig. 2:**
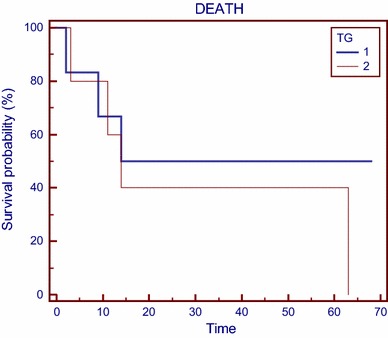
Kaplan–Meier survival curve in patients divided to groups 1 and 2 according to Tg level (TG—thyroglobulin group 1—Tg < 150 ng/mL, and group 2—Tg > 150 ng/mL); time measured in months from the first course of PRRT

Median survival measured from the first course of PRRT was 60 months in group 1 and 17 months in group 2. The difference was statistically non-significant (*p* = 0.475)—data presented at Fig. 2.

### Toxicity

No hypersensitivity reaction was observed during and after infusion of ^90^Y-DOTA-TOC. Moreover, no allergic or other adverse events (like nausea or vomiting) were observed due to the application of amino acid solution for nephroprotection.

No significant increase of creatinine or decrease of glomerular filtration rate was observed during treatment and 3 weeks after completion of PRRT. At later evaluation, 1 year after completion of PRRT, renal toxicity grade 2 was observed in two out of five eligible patients.

With regard to haematological toxicity, a decrease of haemoglobin concentration was observed in all the patients during and after PRRT. The decrease was moderate and haemoglobin concentration did not fall below the limit of 10 mg/dL (toxicity grade 1). Haemoglobin concentration did not improve at 1 year post PRRT. Moreover, in one patient, further decrease of haemoglobin (toxicity grade 2) was found in one patient (no. 5).

Granulocytopenia of grade 1 was observed in two patients. It did not require any therapy and it was not observed any longer 1 year post PRRT. Thrombocytopenia of grade 1 was also noted in one patient—this effect was transient and recovered spontaneously as well. These results are summarized in Table 3.

**Table 3:** Renal and haematological toxicity grades (according to CTCAE Common Terminology Criteria for Adverse Events (2010) version 4.0) in six patients who were treated with all four courses of PRRT

No.	3 weeks post therapy				1 year post therapy			
	Crea	WBC	Hgb	Plt	Crea	WBC	Hgb	Plt
1	0	0	1	0	2	0	1	0
2	0	0	1	0	0	0	1	0
3	0	1	1	1	0	0	1	0
4	0	0	1	0	0	0	1	0
5	0	1	1	0	2	0	2	0
6	0	0	1	0	–	–	–	–

*Crea* creatinine, *WBC* white blood cells, *Hgb* haemoglobin, *Plt* platelets

## Discussion

Treatment possibilities in patients with DTC and non-iodine avid metastases are limited. Therefore, new treatment modalities should be taken into consideration. In this retrospective study, radionuclide therapy with 90-yttrium-labelled somatostatin analogue, ^90^Y-DOTA-TOC was analysed as a non-standard treatment in a small group of patients with non-iodine avid metastases of DTC. This experimental therapy was initiated in 2004, when no other treatment possibility for this small proportion of patients with radioiodine-refractory DTC was available in our centre. Since the overall prognosis for this subgroup of patients is poor, and other options, as chemotherapy or external beam radiation therapy had been known to be ineffective (Cooper et al. 2009; Santini et al. 2002), the newly available PRRT, that was originally designed for patients with disseminated NET, seemed to be an interesting alternative. Evidence of the expression of SSTR in DTC cells (Gabriel et al. 2004; Reubi et al. 1990; Reubi 2003) was convincing enough to support the idea of exploring the feasibility, tolerance and outcome of PRRT in the group of patients with advanced DTC.

In this study, the group of 11 patients was treated with ^90^Y-DOTA-TOC. Due to the highly advanced DTC already at the time of inclusion, despite theoretical life expectancy of above 6 months at the time of inclusion, five patients died before achieving the total planned cumulated activity of 14.4 GBq. Only in one case, death was directly caused by concomitant disease (pulmonary embolism). Patients were monitored for possible adverse events, especially with regard to haematological and renal toxicity. Since no severe adverse reaction to therapy was noted in these major areas of toxicity, we may be certain that the deaths were caused by the disease itself rather than by the applied therapy. This observation is supported by a wide range of publications that confirmed safety of PRRT in large cohorts of patients with NET (Bodei et al. 2012; Imhof et al. 2011).

Of six evaluable patients who received all four courses of PRRT, only one showed signs of partial remission that persisted at least 1 year after this treatment. Two other patients showed both morphological and biochemical stabilization of the disease. The remaining three patients had either overt progression or a combination of stabilization and progression dependent on the method of response evaluation. Similar results were reported in other studies performed on patients with DTC. Gorges et al. (2001) treated three patients with HCTC using 1.7–9.3 GBq of ^90^Y-DOTA-TOC. Progression of the disease could not be stopped in none of the cases. The same compound was applied to seven patients with DTC (four patients with PTC, three patients with FTC) by Waldherr et al. (2001). In contrast to our study, courses of PRRT were applied every 6 weeks. The authors reported stabilization in two patients and progression in the remaining ones. The most recent report by Budiawan et al. (2014) presents results of PRRT using ^90^Y-DOTA-TATE and/or ^177^Lu-DOTA-TATE in 11 evaluable patients with DTC or medullary thyroid cancer that were similar to those obtained in our study: PR was achieved in two patients, SD in four patients, while in the remaining five patients the disease remained progressive. The mean overall survival, however, was much longer in these patients (50 months vs. 21 months in our study) that may be attributed to the disease stage and type (majority of treated subjects had medullary thyroid cancer). The largest group of points with DTC treated with ^90^Y-DOTA-TOC was reported by a group from Basle. Iten et al. (2009) observed biochemical remission expressed by a decrease of Tg in 7 out of 24 patients (29 %). These patients, who had responded to PRRT, did also profit from improved survival time in comparison to those who had not shown any decrease of Tg concentration.

It may be concluded from both, earlier reports and our study, that PRRT shows various results in individual patients with advanced DTC. In general, partial remission or stabilized disease is obtained in about half of the treated subjects. This observation should not be treated as evidence of treatment efficacy as no control group was evaluated in any of published reports. Similarly unequivocal results of PRRT have been reported in patients with NET by several centres (Bodei et al. 2004; Kunikowska et al. 2011).

While discussing the possible causes of relatively poor outcome of the selected treatment method, special attention should be paid to the characteristics of the used somatostatin analogue and the SSTR profile in DTC. Several somatostatin analogues have been developed and studied in different clinical settings. These analogues show different affinity profiles with regard to SSTR subtypes. As reported by Reubi et al. (2000), ^90^Y-DOTA-TOC shows strong binding affinity for the SSTR-2 receptor subtype and weaker affinity for SSTR-3 and SSTR-5. According to some in vitro studies, SSTR-2 is the predominant subtype in DTC tissue. Different SSTR subtypes have been identified in thyroid cancer tissue in vitro. SSTR-2A subtype was expressed in 66 % and SSTR-2B in 83 % of DTC tissue specimens studied with immunohistochemical staining (Atkinson et al. 2013). Klagge et al. (2010) investigated mRNA expression of different SSTR subtypes in thyroid cancer cells. Predominant expression of SSTR-2 and SSTR-5 and a weak expression of SSTR-1 and SSTR-3 were found in DTC cells. Compared to normal thyroid tissue, SSTR-2 and SSTR-3 were significantly upregulated in PTC and SSTR-5 mRNA expression was increased in both, PTC and FTC. These findings are consistent with positive SRS in majority of disseminated DTC cases (Gabriel et al. 2005; Krenning et al. 1993; Pettinato et al. 2008) and also support the promise of effective PRRT, in which SSTR-2 subtype plays crucial role. However, it must be pointed out that the cited in vitro studies were performed on the tissue specimens of uncomplicated cases of DTC. Patients who were qualified to our study had been treated with repeated doses of radioiodine for several years before the dedifferentiation occurred and PRRT was proposed. This long process may have led to changes of SSTR subtypes profile in the DTC cells that we are not aware of, causing lower affinity of the ^90^Y-DOTA-TOC. The problem might be solved in the future with the advent of individualized treatment methods (theranostics), in which appropriate mapping of SSTR subtypes with the use of in vitro or imaging methods shall aid to select the most efficient receptor ligand for PRRT in each patient (Baum et al. 2012).

The suboptimal efficacy of the treatment patients with DTC may be also attributed to dosimetric factors that have not been studied here. It could be of value to investigate SSTR ligands labelled with lutetium-177 that was found to be more effective than yttrium-90 in treatment of smaller lesions (Kam et al. 2012).

The most important limitation of the presented report is the small number of patients. Patients with DTC and non-iodine avid metastases were qualified to PRRT after gaining some positive experience with PRRT in a group of patients with NET. On the other hand, due to the unsatisfactory results of PRRT together with the initiation of clinical trials evaluating tyrosine kinase inhibitors, recruitment of next DTC patients to the latter investigations was favourised (Tai and Poon 2010). Enrolment to the new international trials resulted in rapid decrease of patients’ qualification to PRRT. The low number of cases did not allow for any reasonable statistical analysis. Therefore, the presented paper should be treated as an observational report rather than a proper clinical study.

Heterogenecity of the studied population with regard to DTC histology, age and previous therapy is another limitation of the study. Obviously, it is related to the restricted number of subjects and it could not be avoided.

Another limitation of the study is lack of dosimetric evaluation that could provide some explanation to the heterogenic response pattern to PRRT. Individual analysis of the only patient who achieved partial remission did not explain which clinical, histological or immunological factors were responsible for the favourable outcome. In other words, it was impossible to define predictive factors of response or no-response. It may be hypothesized that the positive response could be attributed to longer effective half-life of ^90^Y-DOTA-TOC in the lesions and, subsequently, high effective dose. This theory would require dosimetric verification, but unfortunately dosimetry in PRRT with 90-yttrium-labelled compounds is troublesome due to pure beta-emission of this nuclide (Hindorf et al. 2007).

Despite some limitations, it may be concluded from our study that some patients with advanced non-iodine avid DTC may profit from PRRT using ^90^Y-DOTA-TOC. As this therapy is generally well-tolerated and the adverse effects are rare and transient, in perspective, PRRT remains an attractive option, especially if individualized forms of PRRT become available to improve treatment outcome.
